# Synthesis, Crystal Structures and Anticancer Studies of Morpholinyldithiocarbamato Cu(II) and Zn(II) Complexes

**DOI:** 10.3390/molecules25163584

**Published:** 2020-08-06

**Authors:** Peter A. Ajibade, Fartisincha P. Andrew, Nandipha L. Botha, Nolwazi Solomane

**Affiliations:** School of Chemistry and Physics, University of KwaZulu-Natal, Private Bag X01, Scottsville, Pietermaritzburg 3209, South Africa; 217067036@stuukznac.onmicrosoft.com (F.P.A.); 217075510@stuukznac.onmicrosoft.com (N.L.B.); 218039521@stu.ukzn.ac.za (N.S.)

**Keywords:** Cu(II), Zn(II), morpholinyldithiocarbamate, crystal structures, anticancer studies

## Abstract

Cu(II) and Zn(II) morpholinyldithiocarbamato complexes, formulated as [Cu(MphDTC)_2_] and [Zn(μ-MphDTC)_2_(MphDTC)_2_], where MphDTC is morpholinyldithiocarbamate were synthesized and characterized by elemental analysis, spectroscopic techniques and single-crystal X-ray crystallography. The molecular structure of the Cu(II) complex revealed a mononuclear compound in which the Cu(II) ion was bonded to two morpholinyl dithiocarbamate ligands to form a four-coordinate distorted square planar geometry. The molecular structure of the Zn(II) complex was revealed to be dinuclear, and each metal ion was bonded to two morpholinyl dithiocarbamate bidentate anions, one acting as chelating ligand, the other as a bridge between the two Zn(II) ions. The anticancer activity of the morpholinyldithiocarbamate ligand, Cu(II) and Zn(II) complexes were evaluated against renal (TK10), melanoma (UACC62) and breast (MCF7) cancer cells by a Sulforhodamine B (SRB) assay. Morpholinyldithiocarbamate was more active than the standard drug parthenolide against renal and breast cancer cell lines, and [Zn(μ-MphDTC)_2_(MphDTC)_2_] was the most active complex against breast cancer. The copper(II) complex had a comparable activity with the standard against renal and breast cancer cell lines but showed an enhanced potency against melanoma when compared to parthenolide.

## 1. Introduction

Cancer is the second leading cause of death globally and continues to be a threat to public health worldwide [1]. Chemotherapy is an effective and potent approach to treating cancer [2], but traditional chemotherapy leads to serious side effects, such as drug resistance and a lack of selectivity where the anticancer drug affects normal body cells in addition to the tumor cells [3]. Although more than sixty drugs have been registered in the USA for the treatment of cancer, there is still an increase in annual cancer deaths; thus, the need to develop new chemotherapeutics agents is of paramount importance [4]. In the search for novel pharmaceutical agents, the use of metal complexes has received tremendous attention [5,6]. This includes the development of cisplatin and its analogues to treat solid tumour cells. Although the use of platinum compounds is acceptable for cancer treatment, it is associated with several side effects [7,8]. The side effects from cisplatin and its derivatives, such as carboplatin and oxaloplatin [9], include ototoxicity, nephrotoxicity, nausea, neurotoxicity and vomiting. These side effects limit the amount of dosage used for treatment [10]. In the search for metal complexes with a novel pharmacological basis, metal complexes of dithiocarbamates are being explored. Their anti-cancer activities are attributed to their ability to form a complex with tumour cellular copper to inhibit the proteasome and, in turn, to initiate tumor cell-specific apoptosis [11,12]. Different metal complexes of dithiocarbamates with promising anticancer activities have been studied [13,14,15]. The mechanism of action of the studied metal dithiocarbamate complexes is similar to that of *cisplatin*, as they strongly and irreversibly bind to DNA [16,17,18]. They also show a better activity on cisplatin-resistant cancer cell lines [19].

Morpholine-based compounds are emerging as a new class of compounds showing outstanding cytotoxic properties, and some of their metal complexes have been evaluated as potential antitumor agents [20,21,22]. Morpholine, a six-membered heterocyclic with both amine and ether functional groups, is viewed as a good building block in medicinal chemistry. The oxygen atom present in its structure is the main reason behind its bioactivity and interactions as a donor–acceptor molecule through which it forms strong complexes with its targets [23,24,25]. The pharmacophoric activity of morpholine is mostly due to the oxygen donor atom, which reduces the basicity of the nitrogen site [26,27,28].

Metal complexes of morpholine dithiocarbamate have received attention in the search for a novel anticancer drug [29,30,31]. Stasevich and colleagues compared the modeled and experimental biological activities of different dithiocarbamates and found that the presence of a morpholine ring in some compounds led to increases in cell inhibition [29]. In the antibacterial studies of nickel(II) and copper(II) morpholine dithiocarbamate complexes, the morpholine dithiocarbamate ligand showed a more enhanced activity than the metal complexes did [30]. Balakrishnan et al. evaluated the antibacterial, antifungal and anticancer activities of some metal complexes of morpholine dithiocarbamates and diamines, which showed poor antibacterial and antifungal properties with the exception of the zinc and copper complexes, which showed very promising antifungal activities and a potent anticancer activity [31]. In other study, zinc dithiocarbamate complexes induce morphological changes in cancer cells and cause apoptosis [32]. In the past, we studied the anticancer potency of copper(II), zinc(II) and platinum(II) of bis-(*N*-methyl-1-phenyldithiocarbamato) complexes that showed a promising anticancer activity [33]. In this study, we report the synthesis, crystal structures and anticancer studies of zinc(II) and copper(II) complexes of morpholine dithiocarbamate.

## 2. Results and Discussion

### 2.1. Syntheses

**Scheme 1:**
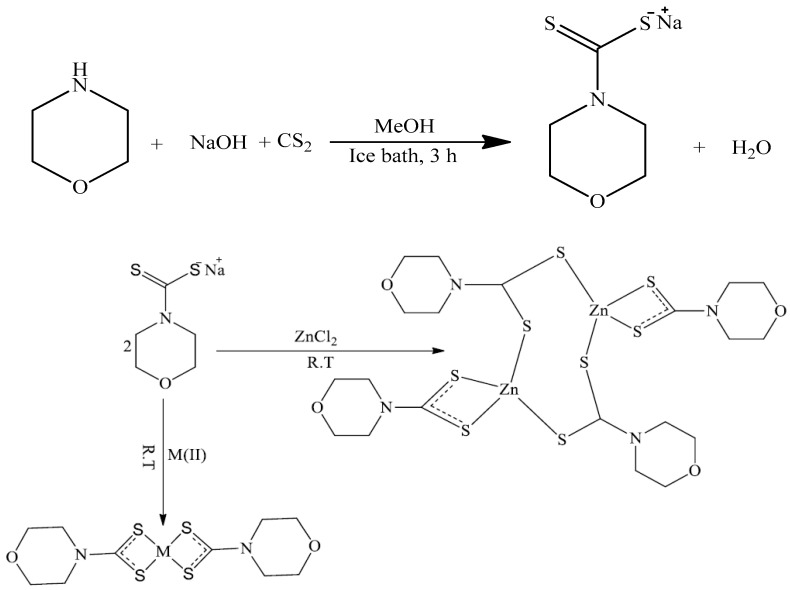
Synthesis of morpholinyldithiocarbamate and the Cu(II) and Zn(II) complexes.

The morpholine dithiocarbamate was prepared in a high yield via the reaction of morpholine dithiocarbamate with carbon disulphide in the presence of sodium hydroxide. The Cu(II) and Zn(II) complexes were obtained from the reaction of the aqueous solution of the metal salt and the morpholinyl dithiocarbamate ligands (Scheme 1). Single crystals of the complexes [Cu(MphDTC)_2_] and [Zn_2_(μ-MphDTC)_2_(MphDTC)_2_] were obtained by slow evaporation of the dichloromethane solution of each complex.

### 2.2. Spectral Studies of the Morpholine Dithiocarbamate and Its Cu(II) and Zn(II) Complexes

The Fourier-transform infrared spectroscopy (FTIR) spectra of the morpholine dithiocarbamate ligand and corresponding Cu(II) and Zn(II) complexes (Figure S1) were carefully compared and assigned. The important regions in the dithiocarbamate metal complexes are the thioureide C–N and the C–S stretching vibrations [34]. In the free morpholine dithiocarbamate ligand, the C–N stretching vibration observed at 1414 cm^−1^ shifted to 1478 cm^−1^ and 1431 cm^−1^ in the Cu(II) and Zn(II) complexes, respectively. These shifts could be ascribed to the coordination of the ligand to the metal ions, causing an electron delocalization within the dithiocarbamate moiety [35,36]. The C–S and C=S stretching vibrations observed as double bands in the ligand in the range of 1108–972 cm^−1^ appeared as single bands at 1007 cm^−1^ and 992 cm ^−1^ in the spectra of Cu(II) and Zn(II), respectively, confirming the coordination of morpholinyl dithiocarbamate to the metal ions as bidentate chelating ligands, in agreement with the single-crystal X-ray structures [31]. The ^1^H-NMR spectrum of the ligand (Figure S2) showed heterocyclic ring protons at 3.77 ppm (N-CH_2_) and 4.36 ppm (O-CH_2_) as triplets. In the Zn(II) complex, the protons appeared upfield upon coordination at 3.67 ppm and 4.04 ppm. In the ^13^C-NMR spectrum of the ligand (Figure S2), the quaternary thioureide (CS_2_) carbon appeared at 208.36 ppm but shifted upfield to 204.04 ppm in the Zn(II) complex (Figure S3). The heterocyclic ring carbons, which appeared at 51.40 ppm and 66.13 ppm in the morpholine ligand, shifted upfield to 51.59 ppm and 66.08 ppm in the Zn(II) complex. The electronic spectrum of the free ligand (Figure S4) exhibited two absorption bands, at 263 nm and 286 nm, attributed to the π-π^*^ transition of the N–C=S and S–C=S [37] In the complexes, the Cu(II) complex exhibited two bands, the intense band at 276 nm attributed to intra-ligand charge transfer transitions mainly associated with N–C=S and S–C=S moieties, and a broad absorption band at 441 nm assigned to the *d-d* transition for copper(II) in a square planar geometry [38]. The electronic spectrum of the Zn(II) complex showed one broad band at 346 nm that resulted from its *d^10^* configuration, attributed to the intra-ligand charge transfer transition [39].

### 2.3. Molecular Structures of the Cu(II) and Zn(II) Morpholinyldithiocarbamate Complexes

The crystal data and structure refinements for both complexes are summarized in Table 1. Selected bond lengths and angles for the compounds are listed in Table 2.

**Figure 1:**
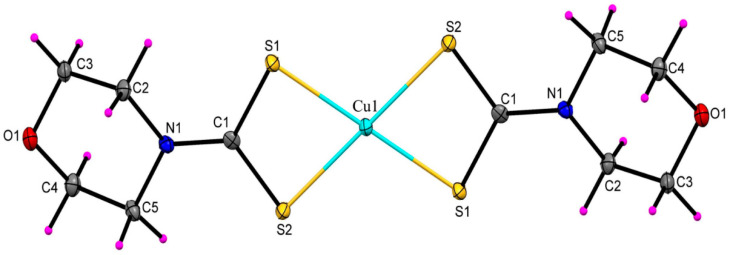
Molecular structure of [Cu(MphDTC)_2_] showing an atomic labeling displacement ellipsoid at a 50% probability.

**Figure 2:**
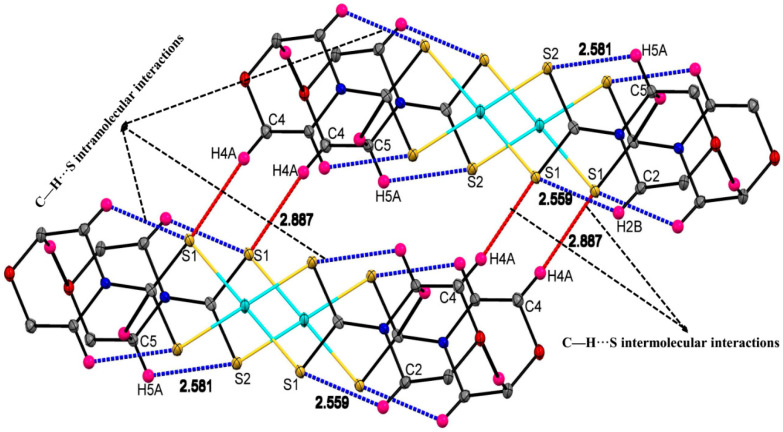
Intra (blue dash lines) and intermolecular (red dash lines) C–H···S interactions of [Cu(MphDTC)_2_]. Non-interacting hydrogen atoms are omitted for clarity.

Horgath and Faulkner [40] reported a Cu(II) morpholine dithiocarbamate complex with a comparable centrosymmetric structure. This polymorph crystallizes in a monoclinic space group P21/c and shows a different crystal packing from the present one. The present complex (Figure 1) crystallized out in a monoclinic space group *P*2_1_/n, and two molecules of morpholinyl dithiocarbamate ligand coordinated the Cu(II) ion as bidentate chelating ligands in a centrosymmetric fashion to form a slightly distorted square planar geometry. The S1–Cu1–S2^1^, 77.22(2)° bite angle is significantly smaller than that of a perfect four coordinate Cu(II) in a square planar geometry [41]. The bond lengths of 2.3019(7) Å for Cu1–S1 and 2.3119(8) Å for Cu1–S2 are typical bond lengths of bis(dithiocarbamato)Cu(II) compounds in a square planar geometry [42]. The bond lengths and angles of the compound are similar to those of the reported Ni(II) morpholine dithiocarbamate complex [43]. The crystal packing for the Cu(II) complex is sustained in the unit cell by inter and intramolecular C–H···S (Figure 2), in addition to *van der Waals* forces.

**Figure 3:**
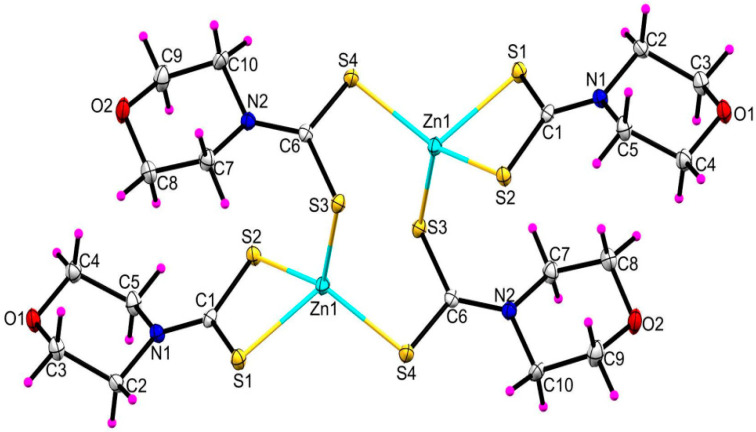
Molecular structure of [Zn(μ-MphDTC)_2_(MphDTC)_2_] showing an atomic labeling displacement ellipsoid at a 50% probability.

**Figure 4:**
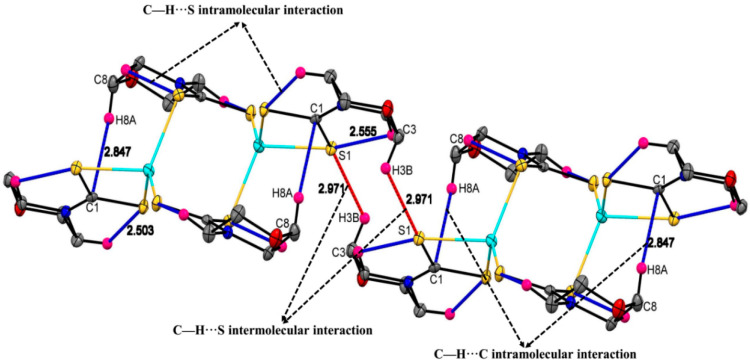
Intramolecular C–H···C, C–H···S (blue dash lines) and intermolecular C–H···S (red dash lines) interactions of [Zn(μ-MphDTC)_2_(MphDTC)_2_]. Non-interacting hydrogen atoms are omitted for clarity.

The Zn(II) complex crystallized in a triclinic space group *P*-1, and only half a molecule was crystallographically independent within the unit cell (Figure 3). In fact, the molecular structure revealed a centrosymmetric dimeric molecule, in which each zinc(II) ion was bonded to one morpholine dithiocarbamate acting as a bidentate ligand with bond lengths of 2.4373(5) Å for Zn1–S1 and 2.4373(5) Å for Zn1–S2. The four-coordinate geometry around each Zn(II) ion was completed by the coordination to two morpholinyl dithiocarbamates acting as bridging ligands between the two Zn(II) ions, with bond distances of 2.342(5)° for Zn1–S3 and 2.3169(5)° for Zn1–S4. The molecular structure of the Zn(II) complex formed a chair-like eight-membered ring [-S-C-S-Zn]_2_ comprised of two zinc(II) ions, two carbon atoms and four sulphur atoms. The Zn–Zn separation of 3.719 Å is similar to other reported dimeric zinc(II) dithiocarbamate complexes [44]. The geometry around each zinc(II) ion was tetrahedrally distorted, with S2–Z1–S3, S3–Z1–S1 and S4–Z1–S bond angles of 115.48, 112.70 and 107.68°, respectively. The bond lengths and bond angles in this compound were in close agreement with reported isostructural dimeric Zn(II) dithiocarbamate complexes [45,46,47]. Each monomeric unit in the crystal packing of the Zn(II) complex was held together by intramolecular C–H···C, C–H···S (blue lines) and intermolecular C–H···S (red lines) interactions (Figure 4).

### 2.4. Anticancer Studies

The anticancer activities of morpholinyldithiocarbamate (Mphdtc) and the Cu(II) and Zn(II) complexes were evaluated against renal (TK10), melanoma (UACC62) and breast (MCF7) cancer cell lines using a Sulforhodamine B (SRB) assay. The results are presented in Table 3. Morpholinyldithiocarbamate was the most active, with an IC_50_ of 1.51 μM against renal cancer and an IC_50_ of 2.65 μM against breast cancer, which was higher than for the standard parthenolide, whose IC_50_ values were 4.64 and 3.52 μM, respectively. The ligand had a comparable activity to the standard parthenolide against melanoma cancer cells. The anticancer activity of the copper(II) complex, [Cu(Mphdtc)_2_], was comparable to that of the standard against renal and breast cancer cell lines, but it was more active against melanoma, with an IC_50_ of 4.47 μM. The zinc(II) complex, [Zn(μ-MphDTC)_2_(MphDTC)_2_], was more active against breast cancer cell lines, with an IC_50_ of 3.17 μM, which was slightly higher than that of parthenolide, which had an IC_50_ of 3.52 μM. The activity of the Zn(II) complex was 8.70 and 16.54 μM against renal and melanoma cancer cell lines, respectively.

## 3. Materials and Methods

### 3.1. Apparatus, Materials and Analysis

The starting materials, copper(II) chloride dihydrate and zinc(II) chloride, were purchased from Sigma Aldrich and used without further purification. The synthesis of morpholine dithiocarbamate ligand (MphDTC) was done using the reported method [48].

^1^H- and ^13^C-NMR spectra were obtained on a Bruker EMX 400 MHz spectrometer (Billerica, MA, USA). The chemical shift values were reported in parts per million (ppm) relative to tetramethylsilane (TMS) as an internal standard. The FTIR spectra of all compounds were recorded on Cary 630 FTIR spectrophotometer (Agilent Technology, Santa Clara, CA, USA) in the 4000–650 cm^−1^ region. Electronic spectra were characterized in DMSO using Cary100 Series UV-Vis spectrophotometer (Agilent Technology, Santa Clara, CA, USA). Elemental analysis was carried out using Thermoscientific Flash 2000 (Thermo Scientific, Waltham, MA, USA). The melting points were recorded using the Stuart^TM^ melting point apparatus (Staffordshire, UK).

Single crystals of the complexes [Cu(MphDTC)_2_] and [Zn_2_(μ-MphDTC)_2_(MphDTC)_2_] were obtained by slow evaporation of the dichloromethane solution of each complex. Suitable crystals (0.34 × 0.22 × 0.14 and 0.33 × 0.22 × 0.14 mm^3^) of Cu(II) and Zn(II) were respectively selected and mounted on a MITIGEN holder in paratone oil on a Bruker APEX-II CCD diffractometer (Billerica, MA, USA). The crystal was kept at *T* = 100(2) K during data collection. Using Olex2 [49], the structure was solved with the ShelXS-2013 [50] structure solution program, using the direct solution method. The model was refined with version 2016/6 of ShelXL [51] using Least Squares minimization.

### 3.2. Synthesis of Bis(Morpholinyldithiocarbamato) Cu(II) Complex [Cu(MphDTC)_2_]

Copper(II) chloride dihydrate (1.066 g, 6.25 mmol) was dissolved in 20 mL of distilled water and added dropwise into a 30 mL aqueous solution of morpholine dithiocarbamate (2.313 g, 12.5 mmol). A dark brown precipitate formed immediately and was further stirred for 1 h at room temperature, filtered, rinsed with methanol several times and dried in a desiccator. Yield 1.632 g, 67%. m.p 307–309 °C. FTIR (cm^−1^): 1007 υ (C-S) cm^−1^ and 1478 υ (C-N) cm^−1^. Anal.Calc (%) for C_10_H_16_CuN_2_O_2_S_4_ (418.89): C, 30.95; H, 4.16; N, 7.22; S, 33.08. Found: C, 30.86; H, 4.13; N, 7.70; S, 32.67.

### 3.3. Synthesis of Bis(Morpholinyldithiocarbamato) Zn(II) Complex [Zn(MphDTC)_2_]

An aqueous solution of zinc chloride dihydrate (0.2726 g, 2 mmol) was added dropwise into a morpholine dithiocarbamate aqueous solution (0.7360 g, 4 mmol) and stirred for 1 h at room temperature. The white precipitate that formed was filtered and rinsed with methanol and was dried in a desiccator to obtain a white solid. Yield 0.4117 g, 53%, m.p 344–346 °C. ^1^H-NMR δ_DMSO-*d*6_: 3.66 ppm (8H, t), 4.04 ppm (8H, t). ^13^C-NMR δ_DMSO-*d*6_: 51.59 ppm, 66.08 ppm and 204.04 ppm. FTIR υ (cm^−1^): 992 υ (C-S) cm^−1^ and 1431 υ (C-N) cm^−1^. Anal. calc (%) for C_10_H_16_N_2_O_2_S_4_Zn (389.89): C, 30.81; H, 4.14; N, 7.19; S, 32.90. Found: C, 30.18; H, 4.14; N, 7.02; S, 33.06.

### 3.4. Anticancer Studies

The growth inhibitory effects of the compounds were tested in the 3-cell line panel consisting of TK10 (renal), UACC62 (melanoma) and MCF7 (breast) cancer cells by a Sulforhodamine B (SRB) assay. The SRB assay was developed by Skehan and colleagues to measure drug-induced cytotoxicity and cell proliferation. The human cell lines TK10, UACC62 and MCF7 were obtained from the National Cancer Institute (Bethesda, Maryland USA) in the framework of a collaborative research program between CSIR and NCI. Cell lines were routinely maintained as monolayer cell cultures at 37 °C, 5% CO_2_, 95% air and 100% relative humidity in RPMI containing 5% fetal bovine serum, 2 mM L-glutamine and 50 µg/mL gentamicin. For the screening experiment, the cells (3–19 passages) were inoculated in 96-well microtiter plates at plating densities of 7–10,000 cells/well and were incubated for 24 h. After 24 h, the cells were treated with the experimental drugs, which were previously dissolved in DMSO and diluted in medium to produce five concentrations. Cells without drugs served as controls. The blank contained complete medium without cells. Parthenolide was used as a standard. The plates were incubated for 48 h after addition of the compounds. Viable cells were fixed to the bottom of each well with cold 50% trichloroacetic acid, washed, dried and dyed by SRB. Unbound dye was removed, and protein-bound dye was extracted with a 10 mM Tris base for an optical density determination at a wavelength of 540 nm using a multiwell spectrophotometer (Agilent Technology, Santa Clara, CA USA). Data analysis was performed using GraphPad Prism software. 50% of cell growth inhibition (IC50) was determined by non-linear regression.

## 4. Conclusions

Copper(II) and zinc(II) complexes of morpholinyldithiocarbamate were synthesized and characterized by single crystal X-ray crystallography. The copper(II) complex crystallized in a monoclinic space group *P*2_1_/n with a Cu(II) ion located on an inversion center, so that the four sulphur donors of the morpholinyl dithiocarbamate ligands were coplanar in a slightly distorted square planar geometry around the copper(II) ion. The Zn(II) complex crystallized in a triclinic crystal system and space group *P-*1, and only half a molecule was crystallographically independent within the unit cell (Figure 3). The molecular structure revealed a centrosymmetric dimeric molecule in which two Zn(MphDTC) units were connected by two bridging morpholine dithiocarbamate ligands. The ligands and corresponding complexes were screened against renal (TK10), melanoma (UACC62) and breast (MCF7) cancer cells by a Sulforhodamine B (SRB) assay. The compounds showed a generally potent activity against the cancer cell lines, with the ligand being the most potent against the renal and breast cancer cell lines. The copper(II) complex was potent against the three cancer cell lines, while the zinc(II) complex was very potent against the renal and breast cancer cell lines. Both complexes showed moderate cytotoxic activity against the melanoma cancer cell lines. The morpholinyldithiocarbamate ligand was more active than the standard drug against the renal and breast cancer cell lines, while the zinc(II) complex showed enhanced activity against breast cancer in comparison to the standard drug. The copper(II) complex was more active than the standard drug against the melanoma cancer cell lines and showed a comparable activity to the standard drug against the renal and breast cancer cell lines.

## Figures and Tables

**Table 1:** Crystallographic data and structure refinements for the complexes.

Compound	[Cu(MphDTC)_2_]	[Zn(μ-MphDTC)_2_(MphDTC)_2_]
Formula	C_10_H_16_CuN_2_O_2_S_4_	C_20_H_32_N_4_O_4_S_8_Zn_2_
*D_calc._*/g cm^−3^	1.806	1.734
μ/mm^−1^	2.113	2.208
Formula Weight	388.03	779.76
Size/mm^3^	0.34 × 0.22 × 0.14	0.33 × 0.22 × 0.14
*T*/K	100(2)	100(2)
Crystal System	monoclinic	triclinic
Space Group	P2_1_/n	*P*-1
*a*/Å	4.1987(5)	7.9581(6)
*b*/Å	20.646(3)	8.7769(6)
*c*/Å	8.3714(10)	11.8945(9)
α/^°^	90	103.680(2)
β/^°^	100.564(8)	91.204(1)
γ/^°^	90	111.329(3)
V/Å^3^	713.39(16)	746.67(10)
*Z*	2	1
*Z’*	0.5	0.5
*Θ_min_*/^°^	1.973	1.778
*Θ_max_*/^°^	26.497	27.412
Measured Refl.	6298	12596
Independent Refl.	1473	3370
Reflections Used	1222	3208
*R_int_*	0.0672	0.0345
Parameters	88	172
Largest Peak	0.496	0.849
Deepest Hole	−0.468	−0.359
GooF	1.088	1.078
*wR_2_* (all data)	0.0930	0.0725
*wR_2_*	0.0873	0.0718
*R_1_* (all data)	0.0448	0.0271
*R_1_*	0.0359	0.0260

**Table 2:** Some selected bond lengths for the complexes.

[Cu(MphDTC)_2_]		[Zn(μ-MphDTC)_2_(MphDTC)_2_]	
**Bond**	**Length(Å)**	**Bonds**	**Length(Å)**
Cu1–S1	2.3019(7)	S1–Zn1	2.4373(5)
Cu1–S2	2.3119(8)	S2–Zn1	2.3347(5)
S2–C1	1.717(3)	S3–Zn1	2.342(5)
O1–C3	1.436(3)	S4–Zn1	2.3169(5)
O1–C4	1.423(3)	C1–S1	1.7290(19)
N1–C1	1.332(3)	C3–O1	1.426(2)
		C8–O2	1.428(2)
		C1–N1	1.323(2)
**Bond**	**Angle(°)**	**Bonds**	**Angle(°)**
S1^1^–Cu1–S1	180.0	S2–Zn1–S1	76.408(16)
S1^1^–Cu1–S2	77.22(2)	S2–Zn1–S3	115.480(18)
S1–Cu1–S2	102.78(2)	S1–Zn1–S3	112.705(18)
S1^1^–Cu–S2^1^	102.78(2)	S1–Zn1–S4	107.689(18)
S1–Cu1–S2^1^	77.22(2)	S2–Zn1–S4	130.37(2)
S2–Cu1–S2^1^	180.0	S3–Zn1–S4	108.203(19)
C1^1^–S1–Cu1	84.58(9)	C1–Zn1–S1	81.75(7)
C1–S2–Cu1	84.37(9)	S2–Zn1–S1	76.408(16)

Symmetry codes, Cu complex: ^1^-x, 1-y, -z; Zn complex: ^1^1-x, 1-y, 2-z.

**Table 3:** Anticancer activities of morpholinyldithiocarbamate (Mphdtc) and the Cu(II) and Zn(II) complexes.

Compounds/Cancer Cells	Renal (TK-10) IC_50_, µM	Melanoma (UACC-62) IC_50_, µM	Breast (MCF-7) IC_50_, µM
Mphdtc	1.51	12.73	2.65
[Cu(Mphdtc)_2_]	4.64	4.47	4.37
[Zn(μ-MphDTC)_2_(MphDTC)_2_]	8.70	16.54	3.17
Parthenolide	4.64	11.37	3.52

## Supplementary Materials

CCDC 1841555 and CCDC 1842399 contain supplementary crystallographic data that can be obtained from the Cambridge Crystallographic Data Centre via www.ccdc.cam.ac.uk/data_request/cif or from The Director, CCDC, 12 Union Road, Cambridge, CB2 1EZ, UK (Fax: +44-1223-336-033; or email: deposit@ccdc.cam.ac.uk.
