# Supplementary material for: Synthesis, Crystal Structures and Anticancer Studies of Morpholinyldithiocarbamato Cu(II) and Zn(II) Complexes
Source: Molecules. 2020 Aug 6;25(16):3584. doi: 10.3390/molecules25163584 (PMC7464096; doi:10.3390/molecules25163584)
Supplement: Supplementary file 1 [file molecules-25-03584-s001.pdf]

molecules

Supplementary Information

Synthesis, Crystal Structures and Anticancer Studies of Morpholinyldithiocarbamato Cu(II) and Zn(II) Complexes

Peter A. Ajibade *, Fartisincha P. Andrew, Nandipha L. Botha and Nolwazi Solomane

School of Chemistry and Physics, University of KwaZulu-Natal, Private Bag X01, Scottsville, Pietermaritzburg 3209, South Africa; 217067036@stuukznac.onmicrosoft.com (F.P.A.); 217075510@stuukznac.onmicrosoft.com (N.L.B.); 218039521@stu.ukzn.ac.za (N.S.)

* Correspondence: ajibadep@ukzn.ac.za

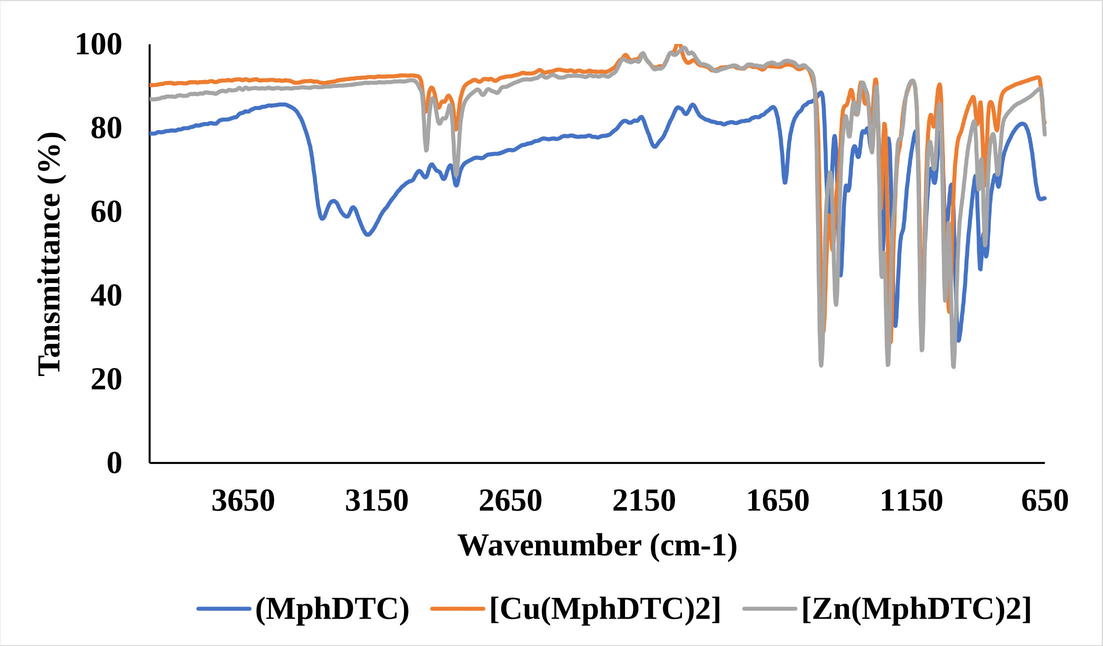

Figure S1. Overlay FTIR spectra of MphDTC ligand, $[Cu(MphDtC)_2]$ and $[Zn(MphDTC)_2]$ complexes.

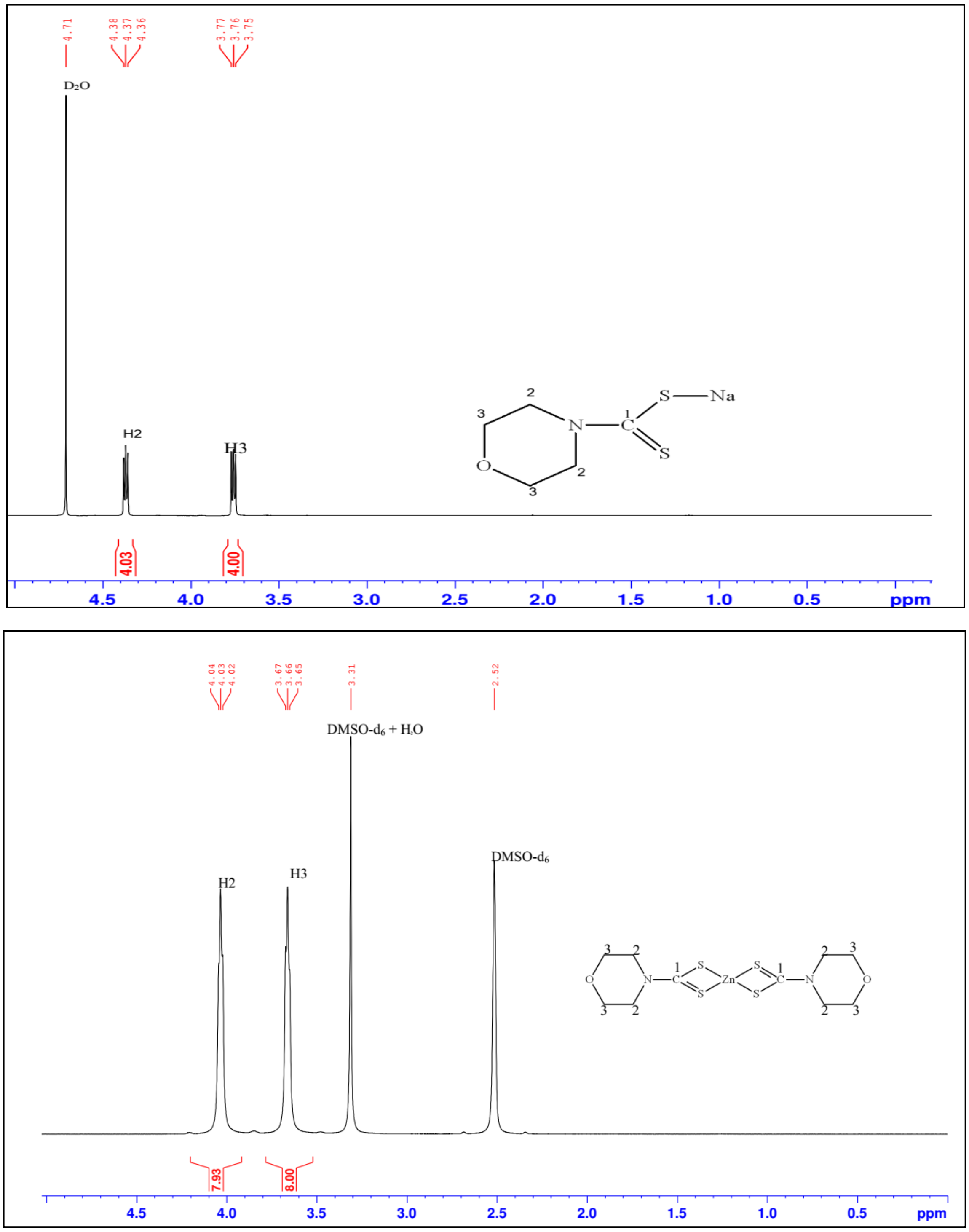

Figure S2. ^{1}H-NMR spectra of morpholine dithiocarbamate ligand and Zn(II) complex.

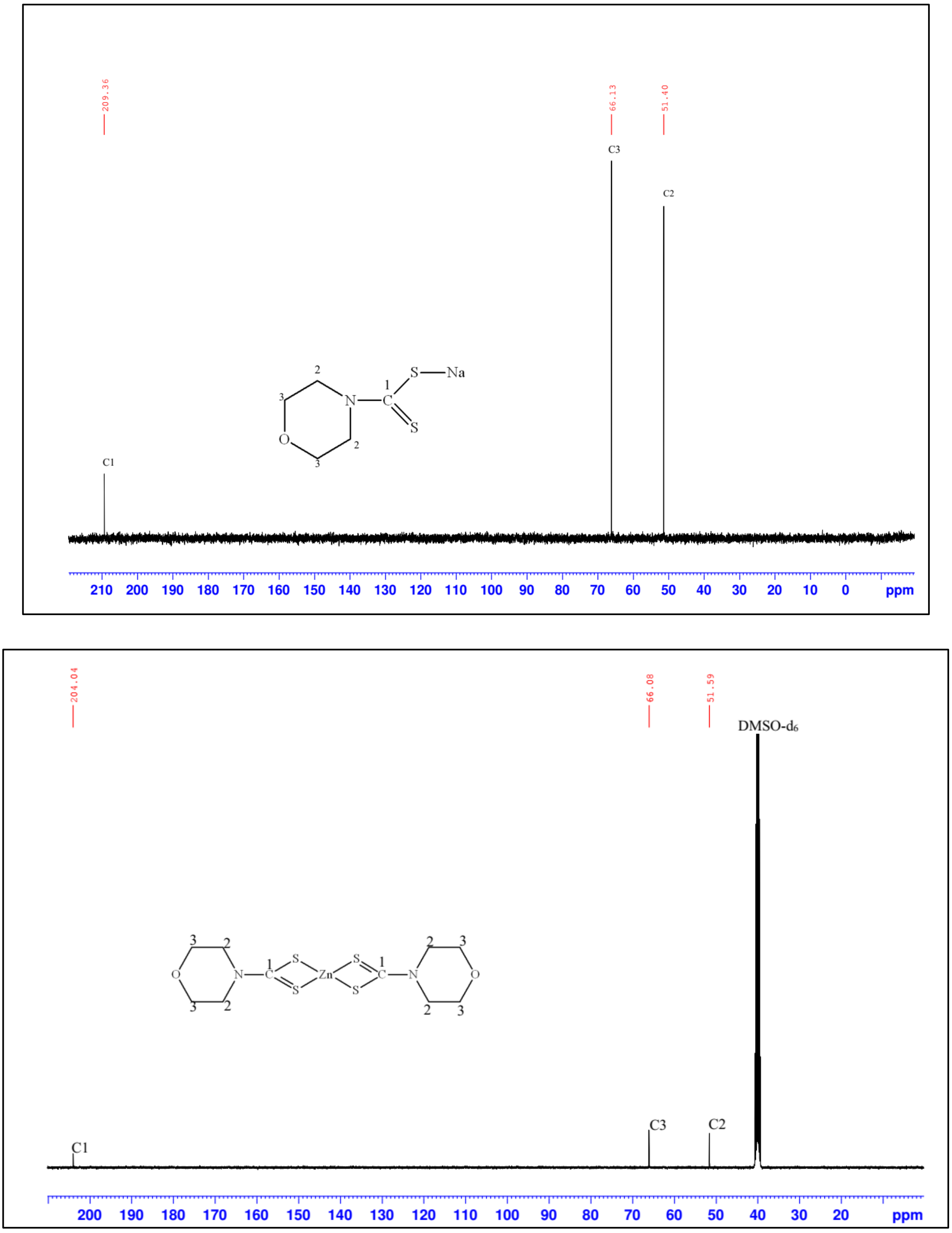

Figure S3. ^{13}C-NMR spectra of morpholine dithiocarbamate ligand and Zn(II) complex.

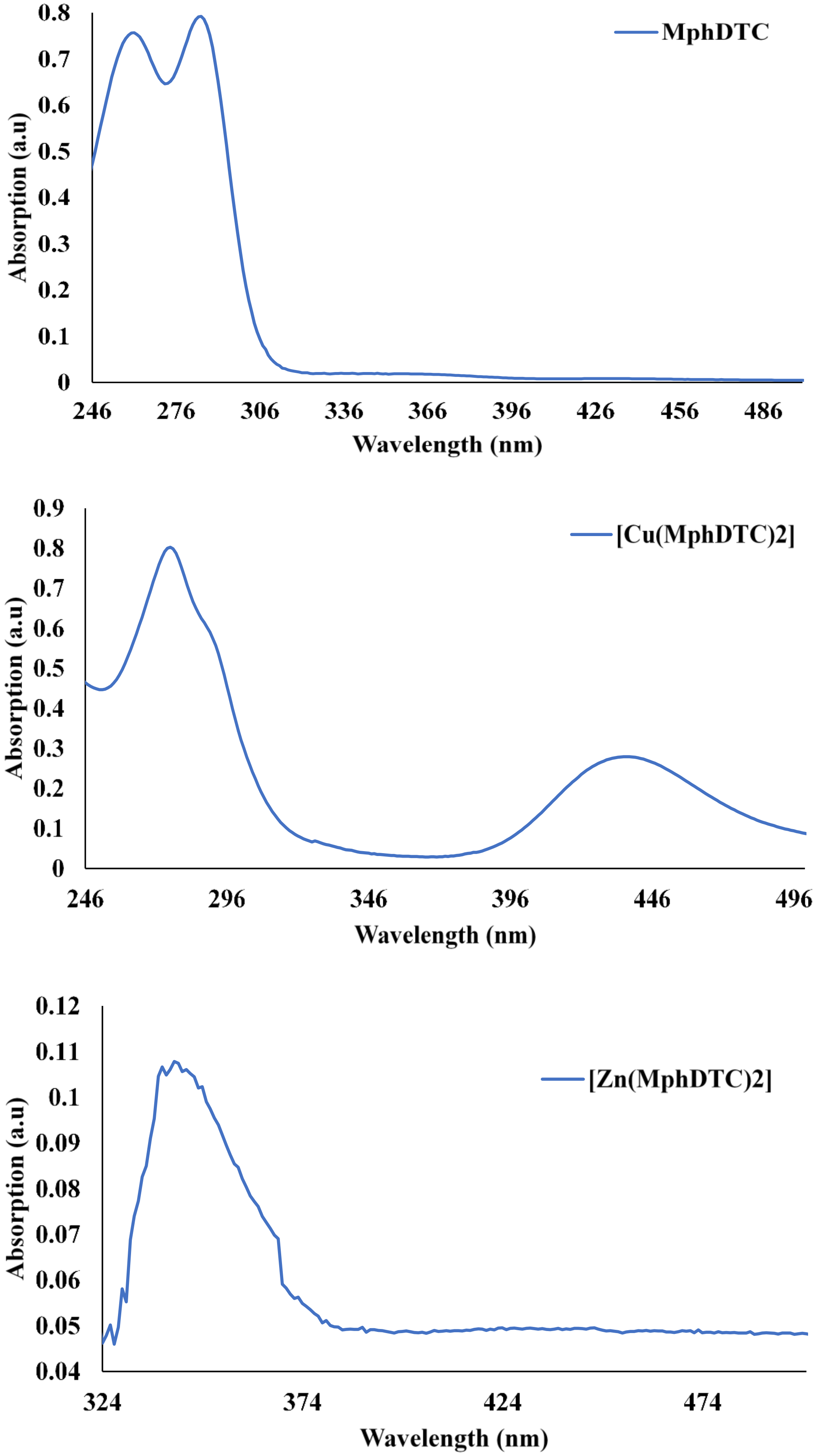

Figure S4. Electronic spectra of morpholine dithiocarbamate ligand and Zn(II) and Cu(II).
